# Evaluation of the Diagnostic Stability of the Early Autism Spectrum Disorder Phenotype in the General Population Starting at 12 Months

**DOI:** 10.1001/jamapediatrics.2019.0624

**Published:** 2019-04-29

**Authors:** Karen Pierce, Vahid H. Gazestani, Elizabeth Bacon, Cynthia Carter Barnes, Debra Cha, Srinivasa Nalabolu, Linda Lopez, Adrienne Moore, Sunny Pence-Stophaeros, Eric Courchesne

**Affiliations:** 1 Department of Neurosciences, University of California, San Diego, La Jolla; 2 Department of Pediatrics, University of California, San Diego, La Jolla

## Abstract

**Importance:**

Universal early screening for autism spectrum disorder (ASD) in primary care is becoming increasingly common and is believed to be a pivotal step toward early treatment. However, the diagnostic stability of ASD in large cohorts from the general population, particularly in those younger than 18 months, is unknown. Changes in the phenotypic expression of ASD across early development compared with toddlers with other delays are also unknown.

**Objectives:**

To examine the diagnostic stability of ASD in a large cohort of toddlers starting at 12 months of age and to compare this stability with that of toddlers with other disorders, such as developmental delay.

**Design, Setting, and Participants:**

In this prospective cohort study performed from January 1, 2006, to December 31, 2018, a total of 2241 toddlers were referred from the general population through a universal screening program in primary care or community referral. Eligible toddlers received their first diagnostic evaluation between 12 and 36 months of age and had at least 1 subsequent evaluation.

**Exposures:**

Diagnosis was denoted after each evaluation visit as ASD, ASD features, language delay, developmental delay, other developmental issue, typical sibling of an ASD proband, or typical development.

**Main Outcomes and Measures:**

Diagnostic stability coefficients were calculated within 2-month age bands, and logistic regression models were used to explore the associations of sex, age, diagnosis at first visit, and interval between first and last diagnosis with stability. Toddlers with a non-ASD diagnosis at their first visit diagnosed with ASD at their last were designated as having late-identified ASD.

**Results:**

Among the 1269 toddlers included in the study (918 [72.3%] male; median age at first evaluation, 17.6 months [interquartile range, 14.0-24.4 months]; median age at final evaluation, 36.2 months [interquartile range, 33.4-40.9 months]), the overall diagnostic stability for ASD was 0.84 (95% CI, 0.80-0.87), which was higher than any other diagnostic group. Only 7 toddlers (1.8%) initially considered to have ASD transitioned into a final diagnosis of typical development. Diagnostic stability of ASD within the youngest age band (12-13 months) was lowest at 0.50 (95% CI, 0.32-0.69) but increased to 0.79 by 14 months and 0.83 by 16 months (age bands of 12 vs 14 and 16 years; odds ratio, 4.25; 95% CI, 1.59-11.74). A total of 105 toddlers (23.8%) were not designated as having ASD at their first visit but were identified at a later visit.

**Conclusions and Relevance:**

The findings suggest that an ASD diagnosis becomes stable starting at 14 months of age and overall is more stable than other diagnostic categories, including language or developmental delay. After a toddler is identified as having ASD, there may be a low chance that he or she will test within typical levels at 3 years of age. This finding opens the opportunity to test the impact of very early-age treatment of ASD.

## Key Points

**Question:**

Is an autism spectrum disorder diagnosis stable by 18 months, the earliest age of American Academy of Pediatrics recommended screening?

**Findings:**

In a cohort study of 1269 toddlers with and without autism spectrum disorder who received their first diagnostic evaluation between 12 and 36 months, overall stability of an autism spectrum diagnosis was 0.84, which was higher than in other groups.

**Meaning:**

Accurate diagnosis of autism spectrum disorder at earlier than 18 months is feasible, and there may be opportunities to test the usefulness of autism spectrum disorder treatment at an early age.

## Teaser

This study examines the diagnostic stability of autism spectrum disorder in a large cohort of toddlers starting at 12 months of age and compares this stability with that of toddlers with other disorders.

## Introduction

Autism spectrum disorder (ASD) is a common disorder of childhood, affecting 1 in 59 children.^1^ It is also becoming clear that ASD has its beginnings during prenatal life.^2^ Because many children with ASD have clinical signs within the first year, such as failure to respond to their name^3^ and reduced positive affect,^4^ there is a considerable demand for early detection, intervention, and services.^5^ Although several studies have shown that early signs of ASD can sometimes be detected using parent report screens as early as 12^6,7,8^ or 18 months of age,^9,10^ the mean patient age at ASD detection is several years later, generally between 3 and 4 years of age.^1^ This late age of detection is a missed opportunity given the accelerated pace of brain development that occurs between birth and 3 to 4 years of age.^11^ Despite the appeal of the concept of early detection and treatment in ASD, there are many unknowns. Foundational questions regarding early-age diagnostic stability, age of clinical symptom onset, and overlap of early-age clinical symptoms between ASD and other disorders, such as language delay or global developmental delay, remain unanswered. A previous report^12^ by the US Preventive Services Task Force did not endorse early universal screening for ASD given the lack of clarity regarding the balance of benefits and harms of early screening and detection.

The months surrounding the first birthday are a remarkable time for a toddler’s development. At this age, toddlers learn to walk,^13^ speak their first word,^14^ and engage in a range of joint social attention behaviors, such as pointing and showing objects to others to share social attentional focus.^15^ The toddler stage is also the earliest age that ASD can be detected and treatment started,^6,16^ yet the stability of an ASD diagnosis at this pivotal age is unknown.

A previous report^17^ stated that most studies examining the diagnostic stability of ASD before 3 years of age have involved slightly older, clinic-referred cohorts, usually at approximately 2 years of age. Stability coefficients within these studies have been high (mean, 88%, range, 63%-100%).^17^ Two studies examined stability at an even younger age (18 months) but examined this question from within multiplex families using the infant sibling design. One of these studies reported that 93% of siblings first diagnosed as having ASD at 18 months retained that diagnosis at a final diagnostic age of 36 months,^17^ but only 69% of siblings first diagnosed as having ASD at 24 months did so (ie, 27 of 39 retained diagnosis).^17^ Although studies collectively suggest that an ASD diagnosis is moderately stable at young ages,^17^ there are several key questions remaining. First, it is unclear whether stability estimates from infant sibling designs would be found within a general population cohort. Second, none of the previous clinic-referred cohort studies included large groups of toddlers without ASD ascertained in the same manner as the toddlers with ASD. Such contrast groups are essential to understand how the ASD phenotype emerges from and overlaps with clinical expressions from other diagnostic groups, such as language and developmental delay, commonly found in clinical settings. Third, clinic-referred studies are small, usually containing 50 to 100 participants, and may generate less stable results. Moreover, children referred to a clinic because of already suspected ASD may generate artificially high stability rates relative to a community-ascertained sample.^17^ Fourth, despite the potential of the infant sibling design to study ASD from birth, stability estimates have only been reported starting at 18 months of age, leaving questions surrounding younger ages unanswered.

Interleaved with these gaps in knowledge is the recent finding from infant sibling studies^17,18^ that 50% to 80% of toddlers eventually diagnosed as having ASD at 3 years of age were not identified as having ASD by expert clinicians at 18 months of age. In short, despite extensive clinical testing that included the gold standard tool the Autism Diagnostic Observation Schedule (ADOS),^19^ these diagnoses were missed. A newer study,^20^ however, suggests that such so-called late-onset cases may be attributable to weaknesses inherent in standardized diagnostic tools at early ages, rather than a lack of observable ASD symptoms per se. Determining the degree to which such late-onset cases may be present in a general population cohort is essential, because if rates are as high as in infant sibling cohorts, it would strongly underscore the American Academy of Pediatrics recommendation for repeat screening at multiple ages. It would also add further urgency to the search for early behavioral or biological tests for ASD to more readily detect ASD during the earliest ages when detection is the most challenging. In this study, we sought to examine the diagnostic stability of ASD in a large cohort of toddlers starting at 12 months of age and to compare this stability with that of toddlers with other disorders, such as developmental delay.

## Methods

### Participants

**Figure 1.**
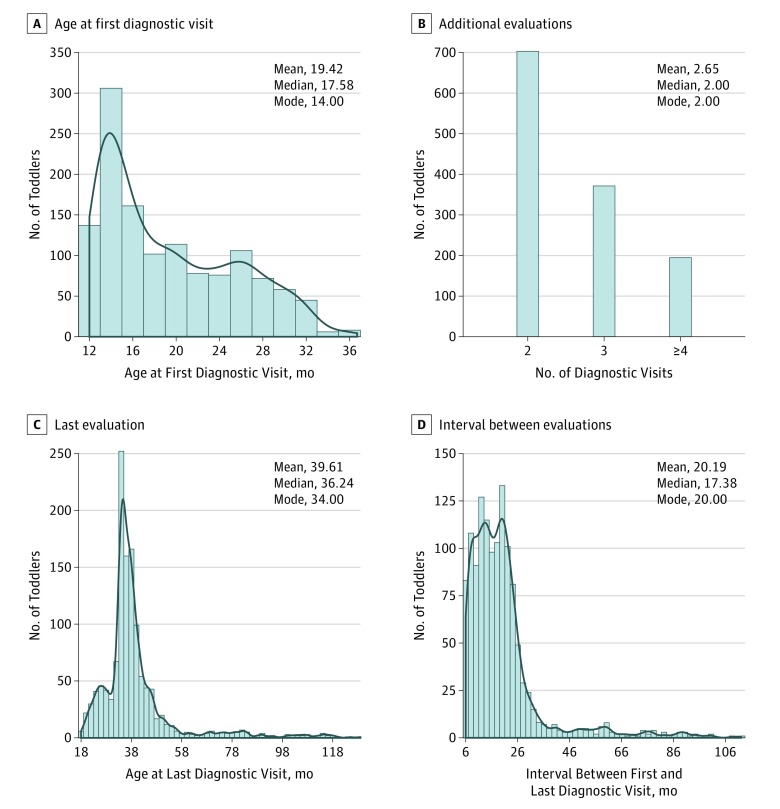
Sample Characteristics Distribution of key features associated with the study cohort, including the age (in months) that toddlers received their first comprehensive diagnostic evaluation (A), the number of toddlers who received 2, 3, or 4 or more diagnostic evaluations (B), the age (in months) that toddlers received their last diagnostic evaluation, and the interval (in months) between a toddler’s first and last diagnostic evaluation (D).

A total of 2241 toddlers 12 to 36 months of age were referred for a diagnostic evaluation to an autism expert evaluation center created at University of California, San Diego. Referrals were given through our early detection program, Get SET Early,^6,21^ which systematically screens for ASD and other disorders in the general population at 12-, 18-, and 24-month well-child checkups or through the general community. Typically developing (TD) toddlers were also recruited from the same pediatric offices participating in the Get SET Early program (eMethods in the Supplement). A total of 1269 of the 2241 toddlers were longitudinally evaluated 2 or more times and were the focus of this study. In this sample, approximately 75% came from the Get SET Early program and approximately 25% from community referral. Additional eligibility requirements included an interval of 6 months or longer between the first and last evaluations. Figure 1 and eFigure 1 and eFigure 2 in the Supplement show the cohort characteristics. This study was overseen by the institutional review board at the University of California, San Diego, and written informed consent was obtained from caregivers before study enrollment. At the data analysis phase of the study, the patient names were removed from our spreadsheets to protect their identity.

### Diagnostic and Psychometric Testing

Highly experienced, licensed psychologists with PhD degrees performed diagnostic and psychometric tests, including the ADOS-2 (module T, 1, or 2 as appropriate),^19^ Mullen Scales of Early Learning,^22^ and Vineland Adaptive Behavior Scales.^23^ Toddlers who received their first diagnostic evaluation at younger than 36 months were diagnostically tested approximately every 12 months until 3 years of age. After each visit, psychologists filled out a diagnostic judgment form and entered it into a database. Psychologists were not masked to previous diagnoses during longitudinal test visits. A toddler was designated as having 1 of the following: ASD, ASD features, developmental delay, language delay (LD), other issue, TD, or typical sibling of an ASD proband. Parents were given feedback regarding their child’s performance after completion of testing and referred for treatment as appropriate. A description of psychologist training, diagnostic criteria used, data quality control process, and estimated Mullen T scores generated for 9% of toddlers who scored below a standard T score of 20 are given in the eMethods in the Supplement. The Table and eFigure 2 in the Supplement give information regarding the *Diagnostic and Statistical Manual of Mental Disorders* (*DSM*) version used.

**Table.** Demographic Information and Clinical Test Scores for Each Diagnostic Group^a^

Characteristic at Last Diagnostic Visit	ASD (n = 441)	ASD Features (n = 78)	DD (n = 89)	LD (n = 80)	Other (n = 91)	Typical Sibling (n = 51)	TD (n = 439)
Sex							
Male	361 (81.9)	68 (87.2)	66 (74.2)	58 (72.5)	61 (67.0)	26 (51.0)	278 (65.6)
Female	80 (18.1)	10 (12.8)	23 (25.8)	22 (27.5)	30 (33.0)	25 (49.0)	161 (36.7)
Age, mean (SD), mo	42.84 (20.28)	40.77 (17.61)	35.91 (10.15)	35.44 (11.42)	42.92 (13.08)	38.44 (13.76)	37.10 (9.84)
Final *DSM* diagnosis^b^							
* DSM-IV*	135	19	24	23	34	26	202
* DSM-5*	306	59	65	57	57	25	237
Ethnicity							
Hispanic/Latino	128 (29.0)	17 (21.8)	36 (40.4)	38 (47.5)	20 (22.0)	15 (29.4)	87 (19.8)
Non-Hispanic/Latino	263 (59.6)	53 (67.9)	47 (52.8)	39 (48.8)	64 (70.3)	31 (60.8)	325 (74.0)
Not reported	50 (11.3)	8 (10.3)	6 (6.7)	3 (3.8)	7 (7.7)	5 (9.8)	27 (6.2)
Race							
White	237 (53.7)	51 (65.4)	48 (53.9)	47 (58.8)	60 (65.9)	31 (60.8)	299 (68.1)
Black/African American	9 (2.0)	1 (1.3)	2 (2.2)	2 (2.5)	5 (5.5)	2 (3.9)	12 (2.7)
Asian	48 (10.9)	7 (9.0)	7 (7.9)	1 (1.3)	2 (2.2)	1 (2.0)	41 (9.3)
Pacific Islander	4 (0.90)	3 (3.8)	4 (4.5)	2 (2.5)	2 (2.2)	1 (2.0)	5 (1.1)
Native American/Alaska	2 (0.50)	0	1 (1.1)	3 (3.8)	1 (1.1)	0	0
Mixed race	57 (12.9)	7 (9.0)	9 (10.1)	1 (1.3)	11 (12.1)	7 (13.7)	46 (10.5)
Not reported	84 (19.0)	9 (11.5)	18 (20.2)	24 (30.0)	10 (11.0)	9 (17.6)	36 (8.2)
Mullen T score, mean (SD)^c^							
Visual reception	38.0 (14.9)	51.4 (13.5)	35.3 (13.4)	49.6 (11.7)	54.1 (13.4)	61.0 (9.7)	59.2 (10.6)
Fine motor	34.0 (12.6)	43.8 (11.6)	31.2 (11.0)	46.4 (10.4)	45.3 (13.2)	53.1 (10.3)	52.4 (10.4)
Receptive language	32.1 (15.0)	46.1 (12.0)	33.4 (12.0)	40.8 (10.6)	48.7 (11.0)	52.7 (10.3)	53.8 (9.0)
Expressive language	30.6 (16.9)	48.6 (12.0)	30.8 (13.9)	33.9 (9.4)	49.2 (12.4)	54.2 (8.8)	53.2 (8.7)
ELC	71.5 (22.1)	94.7 (22.6)	68.6 (17.7)	86.0 (15.1)	99.4 (18.7)	110.5 (14.7)	109.2 (14.4)
Vineland standard score, mean (SD)							
Communication	72.1 (25.0)	96.3 (21.2)	78.3 (21.2)	84.6 (19.5)	98.2 (17.2)	101.2 (18.0)	102.0 (19.4)
Daily living	75.2 (22.5)	95.1 (18.2)	83.8 (19.0)	94.9 (18.6)	96.3 (15.7)	98.7 (16.8)	100.0 (17.7)
Socialization	72.6 (21.5)	95.2 (18.6)	85.9 (18.5)	92.3 (18.4)	97.0 (15.4)	103 (16.9)	102.0 (18.0)
Motor skills	76.2 (27.0)	92.5 (20.4)	80.4 (20.3)	91.9 (23.9)	91.3 (17.9)	95.8 (15.9)	96.3 (19.9)
Adaptive behavior composite	73.3 (21.8)	95.5 (16.2)	80.5 (13.1)	91.23 (11.4)	96.0 (13.4)	100.7 (10.5)	101.6 (12.3)
ADOS (module T, 1, or 2) score, mean (SD)^d^							
ADOS SA/CoSo score	12.9 (4.1)	4.4 (2.7)	3.8 (3.3)	2.4 (2.1)	3.1 (2.4)	2.0 (1.8)	2.2 (1.7)
ADOS RRB score	4.6 (1.9)	2.6 (1.5)	1.4 (1.5)	0.6 (0.9)	0.7 (0.8)	0.3 (0.7)	0.6 (1.0)
ADOS total score	17.6 (4.8)	7.0 (3.1)	5.2 (3.1)	3.0 (2.3)	3.8 (2.6)	2.4 (1.8)	2.8 (2.0)

Abbreviations: ADOS, Autism Diagnostic Observation Schedule; ASD, autism spectrum disorder; CoSo, Communication Social Score; DD, developmental delay; *DSM, Diagnostic and Statistical Manual of Mental Disorders*; ELC, early learning composite; LD, language delay; RRB, restricted and repetitive behavior; SA, social affect; TD, typical development.

^a^ Data are presented as number (percentage) of toddlers unless otherwise indicated.

^b^ Version of the *DSM* used at the final diagnostic evaluation (eMethods and eFigure 2 in the Supplement).

^c^ A total of 9% percent of the overall sample had a chronologic or mental age that exceeded the validated age range for use with the Mullen scales at their last diagnostic evaluation visit and received a *Wechsler Preschool and Primary Scale of Intelligence* instead.

^d^ Administered ADOS module depended on the age and language ability of the toddler at the time of testing. For these individuals, their most recent available Mullen scores were used.

### Statistics and Data Visualization

#### Diagnostic Stability

Stability coefficients were first calculated within 2-month age bands by determining the proportion of toddlers with a particular diagnosis at their first diagnostic visit who retained that same diagnosis at their last visit. Diagnostic transition tables were created for overall and 2-month–interval age-binned data. Diagnostic stability was modeled using logistic regression, with sex, age at first diagnosis, interval between first and last diagnosis, and diagnostic group at first visit as variables and results reported as odds ratios (ORs) (eTable 1 in the Supplement). To examine the association of age at first diagnosis with stability coefficients while optimizing statistical power, we binned age to 4 roughly equally populated groups: younger than 14 months, 14 to 17.99 months, 18 to 23.99 months, 24 months or older. No significant association between sex or interval with stability coefficients was found. Follow-up models with the 4 age bins as the only covariates were used to examine the association of age at first diagnosis with stability coefficients within each diagnostic group. A B-spline method with 3 *df* was also used to test the nonlinear, continuous association of age at first diagnosis with stability coefficients within each diagnosis group and to visualize the data. All analyses were performed in the R programming environment (R Foundation for Statistical Computing) (eMethods in the Supplement). Given caution from the ADOS developers regarding use of the ADOS with toddlers with nonverbal mental ages younger than 12 months and those who are not ambulatory,^24^ stability coefficients were also recalculated after removing such cases.

#### Transition Patterns Visualized Using Diagnostic Heat Maps

To visualize how phenotypic expression of ASD and other disorders changed across visits, diagnostic heat maps were generated using the ggplot library in R software. With the use of this approach, diagnostic judgments were illustrated inward out from first diagnosis to the final diagnosis, and each diagnostic judgment was assigned a unique color. In this way, transition patterns across diagnosis visits could be visually deciphered.

#### ASD Identification Designation and Clinical Differences

For comparison with infant sibling diagnostic stability studies, the ASD cohort was categorized as having an early-age persistent ASD diagnosis, which was defined as an ASD diagnosis at or before 18 months of age that was retained at final diagnosis; middle-age persistent ASD diagnosis, which was defined as an ASD diagnosis after 18 months of age that was retained at final diagnosis; and late-identified ASD, which was defined as ASD not diagnosed at first diagnostic visit regardless of intake age. The 270 toddlers who were initially identified as having TD and retained this diagnosis at final diagnostic age were used as a contrast cohort. One-way analyses of variance with follow-up planned contrasts and Cohen *d* were used to examine clinical differences. Expanded comparisons that included all diagnostic groups were also conducted (eFigure 3 in the Supplement).

## Results

### Participant Characteristics

Among the 1269 toddlers, 918 (72.3%) were male, median age at first evaluation was 17.6 months (interquartile range, 14.0-24.4 months), mean number of diagnostic visits was 2.7 (interquartile range, 2-3), and median age at final evaluation was 36.2 months (interquartile range, 33.4-40.9 months). The Table gives the demographic information and clinical test scores for each diagnostic group.

### Diagnostic Stability

**Figure 2.**
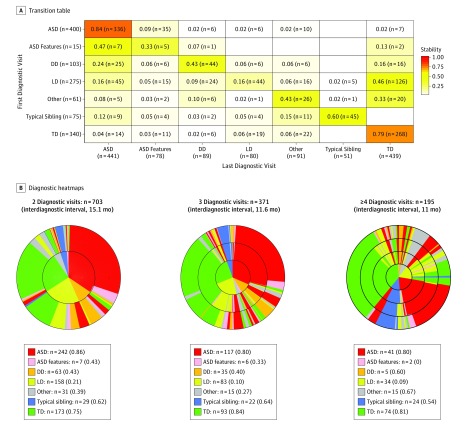
Transition Table and Diagnostic Heat Maps A, Summary of the proportion of toddlers from within the entire sample (N = 1269) who retained or moved to a different diagnostic group between their first and last diagnostic visits. Stability coefficients are denoted within each cell (coefficients are not adjusted for the age at first diagnosis; a high concordance with age-adjusted coefficients was observed) (eTable 4 in the Supplement gives coefficients adjusted for age at first diagnosis). To read the table, compare values across each row or vertically within each column. For example, of the 400 toddlers initially designated as having autism spectrum disorder (ASD), 336 retained this diagnosis at their last (final) diagnostic visit, yielding a diagnostic stability coefficient of 0.84, whereas 35 toddlers had ASD features but no longer met ASD criteria, 6 tested as developmentally delayed, 6 as language delayed, 10 had other developmental issues, and 7 were designated as typically developing with no residual symptoms. For transition tables with stability coefficients within 2-month age bands, see eFigure 4 in the Supplement. B, Changes in diagnosis across visits. Colors represent each diagnostic group and rings represent each diagnostic visit, with the smallest center ring representing the first visit. The left-most panel summarizes diagnostic changes for toddlers who received 2 diagnostic evaluations a mean of 15 months apart; the center represents toddlers who received 3 diagnostic evaluations a mean of 11 months apart; and the right-most panel represents toddlers who received 4 or more diagnostic evaluations a mean of 8 months apart. The heat map indicates that ASD was the most stable diagnostic category, and that toddlers initially suspected as having developmental delay (DD) or language delay (LD) frequently received a final diagnosis of ASD. TD indicates typical development.

**Figure 3.**
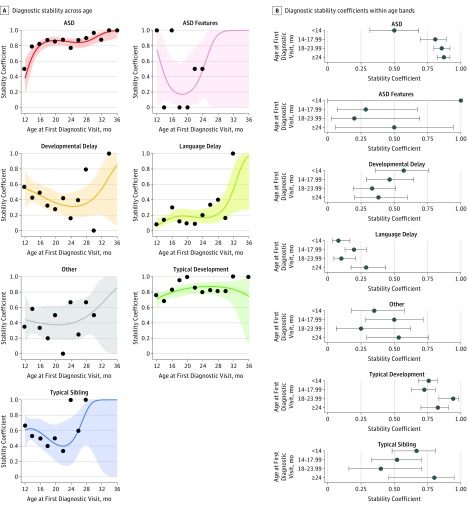
Diagnostic Stability Plots by Age at First Diagnosis A, Plots show diagnostic stability per group across 2-month age intervals based on the age at first diagnostic evaluation. Age intervals with missing data points reflect an absence of toddlers who received their first diagnostic evaluation at that age. B-spline logistic regression line is shown; bands represent 95% CIs for the fit line. Overall stability was highest in toddlers initially designated as having autism spectrum disorder (ASD) or typical development as illustrated by the relatively tight CI bands, and the largely consistent stability coefficients within each age band. B, Diagnostic stability coefficients in the 4 age bins used in the logistic regression model across diagnostic groups. The lines represent 95% CIs. Coefficients were estimated based on within group logistic regression models. eFigure 5 and eFigure 6 in the Supplement give complementary analyses.

Overall stability was 0.84 (95% CI, 0.80-0.87) for an ASD diagnosis and 0.79 (95% CI, 0.74-0.83) for a TD diagnosis (Figure 2A). Results from the overall logistic regression model showed a significant association of age and diagnosis at first visit with stability (eTable 1 in the Supplement). No significant differences were found in stability based on sex (OR, 0.76; 95% CI, 0.56-1.04) or interval between first and last diagnostic evaluations (OR, 0.99; 95% CI, 0.98-1.00). Logistic regression analyses showed a nonsignificant difference in stability coefficients between ASD and TD (OR, 0.86; 95% CI, 0.57-1.29). In contrast, significant differences were found between ASD and the remaining diagnostic groups (OR, 0.11 [95% CI, 0.03-0.32] vs ASD features; OR, 0.15 [95% CI, 0.09-0.25] vs DD; OR, 0.04 [95% CI, 0.03-0.06] vs LD; and OR, 0.16 [95% CI, 0.09-0.28] vs other) (eTable 1 in the Supplement). For ASD, stability was weakest at 12 to 13 months of age (stability coefficient, 0.50; 95% CI, 0.32-0.69). Stability of an ASD diagnosis increased to 0.79 by 14 months of age and 0.83 by 16 months of age (age bands of 12 vs 14 and 16 months; OR, 4.25; 95% CI, 1.59-11.74) (Figure 3 and eFigure 4, eFigure 5, eTable 2, and eTable 3 in the Supplement). When toddlers with ASD features were considered to have ASD, the stability coefficients increased to 0.70 (95% CI, 0.52-0.85) at 12 months of age, 0.85 (95% CI, 0.71-0.94) at 14 months of age, and 0.94 (95% CI, 0.81-0.99) at 16 months of age. Given the transient nature of many early delays,^25^ overall stability was low for the remaining delay groups (Figure 2 and Figure 3 and eFigure 4 and eTable 4 in the Supplement). Exclusion of 73 toddlers (34 with ASD, 1 with ASD features, 24 with DD, 7 with other disorders, 1 with a typical sibling, and 6 with TD) whose nonverbal mental age based on the visual reception component of the Mullen scale was younger than 12 months (mean nonverbal mental age, 9.6 months) did not improve the stability coefficient of ASD at 12 to 13 months (eTable 5 and eFigure 6 in the Supplement).

### Transition Patterns

Diagnostic heat maps (Figure 2B) illustrate diagnostic transition patterns for toddlers who were evaluated 2, 3, or 4 or more times. The transition from an initial diagnosis of LD or developmental delay to ASD was the most common transition type. Transitioning from an initial designation of ASD to a final diagnosis of TD was rare and occurred in only 1.8% of overall cases (ie, 7 toddlers of 400 toddlers initially designated as ASD). However, 5 of these 7 toddlers with false-positive results were initially evaluated at the youngest ages (12-13 months of age) (eFigure 4 in the Supplement).

### ASD Identification Patterns and Clinical Differences

**Figure 4.**
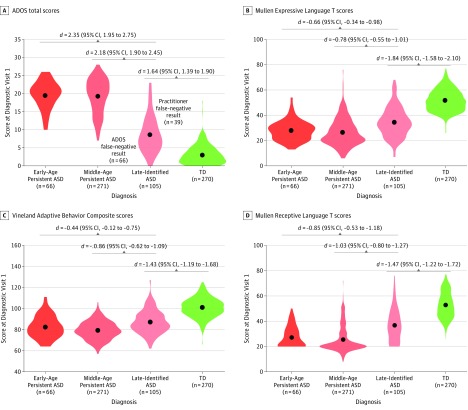
Comparison of Clinical Features in Toddlers With Autism Spectrum Disorder (ASD) Stratified by Identification Age Violin plots show differences in Autism Diagnostic Observation Schedule (ADOS) total scores (A), Mullen Expressive Language T scores (B), Vineland Adaptive Behavior Composite scores (C), and Mullen Receptive Language T scores (D) at the first diagnostic evaluation between toddlers with ASD identified at 12 to 18 months of age (early-age persistent ASD diagnosis), toddlers with ASD identified after 18 months (middle-age persistent ASD diagnosis), or toddlers not identified as having ASD at their first diagnostic visit (late-identified ASD). Black dots represent the mean. The width of the shape represents patient density, and the length illustrates the range of the scores. Data from 270 toddlers with typical development (TD) identified at their first diagnosis visit and retaining that diagnosis at their last visit are shown for comparison. Note that scores from the late-identified group were significantly different from toddlers with TD across all clinical domains, suggesting that symptoms were already present at the first diagnostic visit in a large fraction of late-identified ASD cases. Also note that 39 toddlers in the late-identified group (37%) did fall within the range of concern on the Autism Diagnostic Observation Schedule toddler module (cutoff score for concern using the few to no words algorithm = 10), however, were designated as non-ASD based on practitioner judgment, underscoring the challenges in differential diagnoses particularly at the youngest ages. Effect sizes are reported as Cohen *d* (95% CI). eFigure 3 in the Supplement gives an expanded figure that includes all diagnostic groups.

For ASD, 66 toddlers (15.9%) were classified into the early-age diagnosis group, 270 (61.2%) into the middle-age diagnosis group, and 105 (23.8%) into the late-identified group. Overall, *F* tests revealed a significant between-group difference for all clinical domains (eTable 6 in the Supplement). Follow-up pairwise analyses revealed that these differences were driven by the late-identified group who had consistently better test scores than the other 2 ASD diagnosis groups for all measures at the first evaluation visit (range of Cohen *d* effect sizes, 0.44-2.35). However, toddlers with ASD in this group had significantly worse test scores than toddlers with TD (range of Cohen *d* effect sizes, 1.43-1.84), suggesting that symptoms were present. No clinical differences were found between the early and middle diagnosis groups (Figure 4).

## Discussion

Children with ASD are detected and treated nationally at approximately 4 years of age.^1^ However, we found that within the context of an early detection program,^6^ children can be reliably diagnosed with ASD several years earlier, as young as 14 months. The implications of this finding extend beyond information that relates to diagnostic stability and may open new opportunities to consider if and how treatments started at this early age are associated with long-term outcomes of affected children.

An initial ASD diagnosis was more stable than any other diagnosis, including TD. In our cohort, 84% of toddlers initially diagnosed with ASD at their first visit retained this diagnosis at 3 to 4 years of age. Most toddlers within the remaining 16% did not lose their delays entirely but instead presented with milder delays at their final diagnostic visit. The most common transition was ASD to ASD features, a diagnostic category used for toddlers with signs of ASD but not enough to meet *DSM* criteria. The least common transition was ASD to TD (ie, only 1.8% of toddlers initially designated as having ASD transitioned to TD). Because all toddlers were immediately referred for treatment once any delay was detected, improvements in symptom severity could have been associated with a positive impact of very early treatment, which research suggests may be more beneficial than treatment started at older ages.^26,27,28^ From a public policy perspective, this finding suggests that it is important to initiate treatment immediately after an initial designation of ASD, even at the youngest ages. The human brain has an enormous capacity to resculpt and remodel, particularly during the first postnatal years. The few studies that have examined treatment during this transformative time window have found that toddlers with ASD,^26,27,29,30^ cerebral palsy,^31^ premature birth,^32,33,34^ and severe hearing loss^35^ experience significant positive changes, such as an increase in 15 IQ points^29^ or improvements in speech perception and language ability.^36^ The caveat, however, is that early-age diagnostic evaluations should be conducted by practitioners with considerable experience in early ASD development. In many places in the United States, such experience is severely lacking.^37^

Although the overall stability of an ASD diagnosis was high, examination of the data within 2-month age bands revealed that stability was selectively low at 12 months, with a stability coefficient of 0.50. The lower stability coefficient for ASD specifically at 12 months is likely reflective of some limitations in the diagnostic tools used at that age, which included the ADOS-2 and *DSM*. In the first diagnosis ASD sample, which contained 400 toddlers, only 7 transitioned to a final diagnosis of TD, and 5 of these were within the 12-month age band. Research from the ADOS-2 developers cautions that it is not valid for use with toddlers who have a nonverbal mental age younger than 12 months,^24^ yet even when such toddlers were removed from analyses, the stability coefficient did not improve. Twelve months is an age when toddlers learn to talk, walk, merge, and shift attention with objects and others, and it is not surprising that this age would be the most diagnostically challenging. When toddlers with ASD features were included in the calculation at 12, 14, and 16 months of age, stability coefficients increased to 0.70 at 12 months, 0.85 at 14 months, and 0.94 at 16 months. This finding is likely related to the possibility that ASD is a dimensional rather than categorical disorder^38,39^ and the strict cutoff boundaries defined by the *DSM* may artificially affect results.

Our study also found that toddlers diagnosed as having an LD at their first visit overwhelmingly transitioned into testing within the typical range by 3 to 4 years of age. Such transient LD cases have been commonly noted in the literature.^25^ Toddlers who exhibited an LD were referred for immediate treatment and generally received 1 to 2 hours per week of speech therapy. Our study was not designed to determine whether such early treatments affected the speed with which toddlers caught up by the time they reached final diagnosis age. Another possibility is that the psychometric test that we used (Mullen Scales of Early Learning) may be less reliable at very young ages.

The importance of understanding ASD diagnostic stability in a general population, community-ascertained cohort should not be underestimated, particularly when early screening starting at 18 months is recommended by the American Academy of Pediatrics,^40^ yet most screening studies^9,10,41,42^ validate diagnoses only once, usually at approximately the age of screening, leaving unclear the degree to which an initial diagnosis persists at later ages. Although this was not a screening study and the cohort contained approximately 25% community-referred cases, most of the ASD cases were detected using a broadband screening tool, the Communication and Symbolic Behavior Scales Infant-Toddler Checklist,^43^ administered at well-child visits.

In this study, with a sample size of 1269 toddlers from the general population, each with multiple evaluation visits, generating a total of more than 3000 evaluation visits, we found that 105 (23.8%) who ultimately received a diagnosis of ASD at 3 to 4 years initially had ASD missed at their first evaluation visit. This percentage is substantially lower than the 50% to 80% late-identified ASD cases reported in infant sibling studies.^17,18^ Among the patients with late-identified ASD, 45 (42.8%) were initially suspected of having only an LD. That is, practitioners focused on a child’s delays in language rather than subclinical social delays. This finding is consistent with an infant sibling study^17^ that reported lower-than-expected language scores on the Mullen Scales of Early Learning test within their late-identified group.

### Limitations

One limitation to our study was that the practitioners who made the final diagnoses were not masked to previous diagnoses. This lack of masking was because *DSM-5* procedures and criteria require consideration of historical information regarding ASD symptoms. Although unlikely, it is possible that review of this information could have biased psychologists in favor of increased diagnostic stability. Evidence counter to this point is the relatively weak diagnostic stability found in other delay groups.

## Conclusions

Autism spectrum disorder is a common disorder that begins in prenatal life.^2^ Because of this, there is a demand for early detection, intervention, and services,^5^ and,in response, marked effort and funding have gone into discovery of methods for early-age universal screening, detection, and diagnosis. Therefore, when ASD is not detected in an infant or toddler, it is likely because it was missed.^20^ Our findings suggest that ASD detection and diagnosis can reliably start as young as 14 months. Our next challenge is to determine best treatments and the degree to which such early engagement benefits toddlers and their families in the long term.

## Notes

Supplement. eMethods. Supplementary methods eFigure 1. Examination of clinical characteristics between excluded (1 visit) and included (≥2 visits) toddlers eFigure 2. Distribution of diagnostic judgements based on the DSM version eFigure 3. Clinical characteristics of ASD group stratified by identification age and other DX groups eFigure 4. Diagnostic transition tables within each 2-month age band eFigure 5. Diagnostic stability plots by age at first diagnosis eFigure 6. Diagnostic stability after removing toddlers with non-verbal mental age <12 mo eTable 1. Overall logistic regression model eTable 2. Effect of age at first DX on stability coefficients eTable 3. Stability coefficients within 2 months age bands eTable 4. Overall age adjusted and unadjusted stability coefficients eTable 5. Distribution of toddlers with non-verbal age below 12 mo. Across diagnosis groups eTable 6. Between-group difference for all clinical domains eReferences
