# Supplementary material for: Evaluation of the Diagnostic Stability of the Early Autism Spectrum Disorder Phenotype in the General Population Starting at 12 Months
Source: JAMA Pediatr. 2019 Apr 29;173(6):578–87. doi: 10.1001/jamapediatrics.2019.0624 (PMC6547081; doi:10.1001/jamapediatrics.2019.0624)
Supplement: Supplement. — eMethods. Supplementary methods eFigure 1. Examination of clinical characteristics between excluded (1 visit) and included (≥2 visits) toddlers eFigure 2. Distribution of diagnostic judgements based on the DSM version eFigure 3. Clinical characteristics of ASD group stratified by identification age and other DX groups eFigure 4. Diagnostic transition tables within each 2-month age band eFigure 5. Diagnostic stability plots by age at first diagnosis eFigure 6. Diagnostic stability after removing toddlers with non-verbal mental age <12 mo eTable 1. Overall logistic regression model eTable 2. Effect of age at first DX on stability coefficients eTable 3. Stability coefficients within 2 months age bands eTable 4. Overall age adjusted and unadjusted stability coefficients eTable 5. Distribution of toddlers with non-verbal age below 12 mo. Across diagnosis groups eTable 6. Between-group difference for all clinical domains eReferences [file jamapediatr-173-578-s001.pdf]

Supplementary Online Content

Pierce K, Gazestani VH, Bacon E, et al. Evaluation of the diagnostic stability of the early autism spectrum disorder phenotype in the general population starting at 12 months. *JAMA Pediatr.* Published online April 29, 2019. doi:10.1001/jamapediatrics.2019.0624

eMethods. Supplementary methods

eFigure 1. Examination of clinical characteristics between excluded (1 visit) and included (≥ 2 visits) toddlers

eFigure 2. Distribution of diagnostic judgements based on the DSM version

eFigure 3. Clinical characteristics of ASD group stratified by identification age and other DX groups

eFigure 4. Diagnostic transition tables within each 2-month age band

eFigure 5. Diagnostic stability plots by age at first diagnosis

eFigure 6. Diagnostic stability after removing toddlers with non-verbal mental age <12 mo

eTable 1. Overall logistic regression model

eTable 2. Effect of age at first DX on stability coefficients

eTable 3. Stability coefficients within 2 months age bands

eTable 4. Overall age adjusted and unadjusted stability coefficients

eTable 5. Distribution of toddlers with non-verbal age below 12 mo. across diagnosis groups

eTable 6. Between-group difference for all clinical domains

eReferences

This supplementary material has been provided by the authors to give readers additional information about their work.

eMethods. Supplementary methods

Subject recruitment

The Get SET Early ASD Detection Program (formerly the 1-Year Well-Baby Check-Up Approach)[1]. The Get SET Early model, developed by first author K.P. as part of an NIMH ASD Peds Network Consortium[2], focuses on a three-step process where S=Screen, E=Evaluate, and T=Treat linking the critical elements of screening, evaluation and treatment in a rapid fashion. The overall goal of the model is to detect all cases of ASD within a pediatric office by the second birthday. To support the first step of screening, a local pediatrician network of 170 pediatricians at 40 offices was established and trained to administer a parent report broadband screening tool, the CSBS IT Checklist[3], to all toddlers at 12, 18, and 24 month well baby visits. Pediatricians were provided training on the scoring and interpretation of screening and identification of early developmental delays. Pediatrician office staff were trained to refer any children with developmental concerns based on a failed CSBS or pediatrician concern to our Center for further evaluation. As part of the model, a state-of-the-art evaluation center with autism expert evaluators was established to ensure rapid and accurate assessment for delay. All clinicians were PhD level licensed psychologists with expertise with toddlers with autism. Immediately following the evaluation, test results were reviewed with the parents and referrals were made for appropriate community treatments the same day. Overall, this three-step model allows for a thorough yet rapid process to engage families in early intervention as soon as a delay is detected and very young ages.

Community Self-Referred Sample. Due to our Center's high profile within the community, ~25% of our sample is based on community referrals outside of the Get SET Early Model. Parents of these toddlers sought out a developmental evaluation independent of their pediatrician due to their own concerns about their child's development. In some cases, parents were referred to our Center by professionals in the community (e.g., a speech therapist). Once a connection is established at our Center, self-referred families proceed through the same process of evaluation and referral for treatment as toddlers detected by the Get SET Early Model.

Given that the majority of the sample was ascertained via universal screening wherein all toddlers were screened at well-baby visits, our cohort is likely a valid representation of the general population within San Diego county. Moreover, our unique subject ascertainment approach has resulted in one of the youngest and largest general population samples in the literature to date.

Examining clinical characteristics of included vs excluded toddlers

Psychologists at our Center initially performed developmental evaluations with 2,241 toddlers. Within this sample, 1,269 toddlers were evaluated two or more times and are the focus of this study. Although it is possible that meaningful clinical differences are inherent between toddlers who were excluded from the current study versus those that were included, a lack of obtaining a 2nd evaluation was primarily due to a toddler's age at his/her first diagnostic evaluation. That is, if a toddler was referred at 32 months or older, we did not schedule a follow up evaluation given the considerable expense associated with in-depth diagnostic testing via licensed clinical psychologists. This is evident in our histogram (Figure 1A) that shows that very few toddlers referred at 32 months or older are included in this study (simply because we did not call them back in for an evaluation; not that they dropped or had missing data).

Another reason that some toddlers may not have had a 2nd diagnostic evaluation is due to the fact that they are still waiting for it to occur. Our Center protocol is that toddlers are invited for additional diagnostic evaluations approximately once per year until age 3 years. Thus, a portion of our sample was evaluated within the past 1 year and are awaiting their follow up test visit.

We nonetheless compared the clinical characteristics of ASD toddlers who were excluded from the study (i.e., toddlers that had only one diagnostic evaluation) to toddlers included in the study (i.e., toddlers with two or more diagnostic evaluations) using t-tets. There were no statistically significant differences in clinical characteristics. As expected, however, non-included toddlers were significantly older at their first diagnostic evaluation visit. **See eFigure 1.**

Diagnostic criteria and DSM version

A toddler was designated in each of the following diagnostic categories based on the following criteria: *Autism Spectrum Disorder (ASD)* - scored within the range of concern on the ADOS and was considered ASD based on DSM (DSM-IV or DSM-5) criteria and clinical judgment. *ASD Features* - showed signs of autism and may have an elevated ADOS score, but did not meet full criteria for ASD. *Developmental Delay (DD)* - > 1 standard deviation below expected values on two or more areas of the Mullen with at least one of those areas outside of the verbal scales. *Language Delay (LD)* - > 1 standard deviation below expected values on either or both the receptive

© 2019 Pierce K et al. *JAMA Pediatrics.*

or expressive subtests on the Mullen. *Other* – showed developmental issue not captured in any of the aforementioned categories including motor delay, social emotional delay, attention deficit and speech articulation impediment. Toddlers were determined to be *typically-developing, TD*, if they fell within the normal range on all clinical assessments and *TypSib* if they also had a sibling with ASD. See **Table 1** for subject characteristics based on each toddler's final diagnostic visit.

Data collection for the present study began with small subject samples between 2006-2007, with the bulk of subject data collection occurring between 2008-2018. On May 18th 2013, a new version of the Diagnostic and Statistical Manual (DSM), the DSM-5, was released and our Center adopted the new DSM-5 criteria on August 1st, 2013. Thus, toddlers that participated in all of their evaluations prior to August 2013 were diagnosed using only DSM-IV criteria, and toddlers that participated in all of their evaluations after August 1st 2013 were diagnosed using only DSM-5 criteria. A percentage of toddlers (~20%), began in the study during the time period of the DSM-IV, but ended during the time period of the DSM-5 and thus psychologists used both DSM versions as part of their diagnostic evaluation. **eFigure 2** illustrates the percentage of toddlers within each diagnostic group who received the DSM-IV only, the DSM-5 only, or both. As illustrated, within the ASD group, 52% were diagnosed using DSM-IV criteria only, 31% using DSM-5 only and 17% using both.

Collection of clinical history, parent feedback and treatment referral

Clinical History. The unique age and subject ascertainment procedure used in our study brings with it some variation in the quantity of historical information collected across subjects. With a modal age of 14 months, our cohort is very young and some parents brought their child for an evaluation solely because their pediatrician recommended they do so based on a failure of the screening tool we use in our early detection program, the CSBS, and not because they had any initial concerns. A brief history and parent concerns, if any, are gathered first by study coordinators when the family first contacts our Center, and then reviewed by the psychologist. The psychologist also asks the family about any concerns and past assessments, prior to starting the evaluation session. While the Vineland Adaptive Behavior Scales was collected on all subjects, in some cases, the Vineland was administered as an interview, and in other cases the parents filled out the form. In either case, the Vineland served to provide further history for the psychologist.

Parent Feedback and Treatment Referral. Feedback regarding standardized test results and general clinical impression was provided to parents following completion of testing at every evaluation visit. The child's strengths and weakness were discussed with the parents, and any evidence of developmental delay was reviewed in detail. Based on the delay, the child was immediately referred for appropriate community services (e.g. speech therapy for language delays, ABA based services for delays related to ASD, etc.) with parent consent. Prior to age 3 years, the psychologist would share concerns for delay, and once the child turned 3 years, the psychologist made a formal diagnosis based on the child's current presentation. In our community, showing risk factors for ASD qualifies a child under age 3 years for autism-focused services including ABA-based services which are generally 8-10 hours per week. Therefore, it is standard protocol within our community to wait until age 3 years to make an ASD diagnosis given progressive service availability and given that previous research has shown the diagnosis of ASD is reasonably stable at age 3 years (see[4] for a review). For research purposes, the psychologist completes a diagnostic judgment form following every evaluation visit to track diagnostic impression over time and calculate the stability examined in the current study.

Description of psychologist training and reliability

At any given time across the study period, a total of two or three psychologists performed diagnostic evaluations. One of the diagnosticians, author CCB, achieved research reliability directly with the ADOS creator, Catherine Lord, prior to the study and was responsible for training new psychologists across the entire study period which generally extended across a 3-6 month period. CCB has achieved the highest level of ADOS certification as a certified independent ADOS trainer. Across the study period, inter-rater ADOS reliability between psychologists was established at >.80 on algorithm items across all modules. Across the past year, inter-rater reliability has been performed once every month, for both diagnostic impression and for ADOS algorithm items with 100% concordance in overall diagnosis, and an average of .89 on ADOS algorithm items.

Data verification and QC process

Using scatterplots, data were plotted for subdomain and total scores for each standardized test to visually identify data entry errors. A script was also written that generated a list of subjects whose scores fell outside of the possible range on that test (e.g., ADOS-2 Toddler Module Total score > 28). Data errors, which comprised <1% of

© 2019 Pierce K et al. *JAMA Pediatrics.*

all data, were fixed by retrieving the original test booklet used by the psychologist during testing and re-entering test scores.

Mullen estimated T score

The lowest minimum subscale T score based on the Mullen scoring manual is 20. However, some toddlers in our study performed at levels that were below 20, and we elected to generate a score for each toddler that was an approximate reflection of ability, rather than artificially assigning all such toddlers a score of 20. In these cases, which occurred in 9.4% (120 toddlers) of our sample, we estimated T-scores using the raw scores obtained with the table for the child's chronological age, as follows. The estimated T score is calculated by examining the variation in raw scores for the lowest T scores available for the child's age and applying that variation to estimate a lower T score. For example, if the lowest raw score available for the child's age is 14, but the child actually has a raw score of 12, two steps would be counted down and an estimated score would be calculated based on the amount of difference between T scores for each raw score above the cut off. So, if the raw score of 14 corresponded to a T of 20, and there was a 2 point difference between each T score above 20, then the estimated T score would be 16 (2 steps times 2 point difference = 4 and thus the estimated T score is 20-4 =16).

Diagnostic transition tables per 2-month age band

In addition to the overall diagnostic transition table presented in the Figure 2 that was calculated for the entire 1,269-subject cohort regardless of age at first diagnosis, diagnostic transition tables were also created for each 2-month age band. These tables provide detail regarding the sample sizes per 2-month age band, as well as diagnostic stability coefficients within each age band and diagnostic category. **See *e*Figure 3.**

Employing a linear regression model to assess the effect of age at first DX on stability

In addition to using logistic regression as described in the main body of the paper, we also examined the potential effects of gender, age at first diagnosis, and diagnostic group (based on diagnosis at last visit) on the stability coefficients using a non-parametric linear regression model. To do so, the cohort was partitioned into 9 age bands based on 2-months age intervals up to 30 months (e.g., 12-13 months, 14-15 months etc.) and given the relatively small ASD sample sizes at older ages data from 30-36 months was collapsed into a single age band for a grand total of 10 age bands. Stability coefficients were determined for each combination of age band, gender, and diagnostic group. Linear regression was next used to model stability coefficients with age (mean age within each age band; age effects were modeled with a B-spline method using three degrees of freedom), diagnostic group (7 groups), and gender (male, female) as predictors.

The overall regression model was significant, R^2=.49, p<.0001. There were no significant differences in stability based on sex (overall stability .84 boys; .84 girls (β = .0039, p=.934), thus, all stability coefficient data presented in tables and figures are reported collapsed across sex. Linear regression analyses revealed that stability coefficients were similar between ASD (.84) and TD (.78) p=.775. In contrast, significant differences were found between ASD and the remaining diagnostic groups (all ps <.0016). Within the ASD group, stability significantly changed across ages (X^2=31.01, p=.0003; ***e*Figure 4**). Fisher's exact test revealed that only one age band, 12-13 months, was significantly lower than other age bands (stability coefficient .50, p=.00003; ***e*Figure 5**) and only one age band, 30-36 months, was significantly higher (stability coefficient .94, p=.026). Stability of ASD diagnosis increased to .79 by 14 months, and .83 by 16 months **(*e*Figure 5).** Given the transient nature of many early delays[25], not surprisingly overall stability was low for the remaining delay groups (all coefficients <.50).

Impact of including toddlers with non-verbal age equivalents < 12 months

Developers of the ADOS Toddler Module note that the validity of the test might be weakest at the youngest ages and/or with toddlers who have not yet achieved a non-verbal mental age of 12 months. Luyster and colleagues (2009) state: *"Pilot analyses indicated that children developmentally younger than 12 months consistently obtained elevated scores... We therefore set a lower cutoff of 12 months non-verbal mental age."* Thus, one potential reason that the diagnostic stability was lowest at the youngest age bands, particularly the 12-13 month old age range, is because a percentage of toddlers had a non-verbal mental age that was lower than 12 months at the time of ADOS testing. To examine this possibility 73 subjects were removed from analyses (34 ASD, 1 ASD Features, 24 DD, 7 Other, 1 Typ Sib, and 6 TD) whose age equivalent scores were less than 12 months based on the visual reception subtest score of the Mullen. **See *e*Table 5** for more detail. Instead of improving diagnostic stability coefficients as would be predicted, removing these subjects actually lowered the diagnostic stability coefficients. As such, we do not believe that the lower cutoff of 12 months is required in order to obtain valid ADOS toddler module score. **See *e*Figure 5**.

© 2019 Pierce K et al. *JAMA Pediatrics.*

Early, Middle and Late-Identified ASD in comparison to all diagnostic groups

In the main body of the paper, selected scores from the Mullen, Vineland and ADOS were compared between Early, Middle and Late-Identified toddlers with ASD. Typically developing toddlers that received a diagnosis of TD at every visit were used as a contrast. Here we provide an expanded visualization of clinical scores that includes all study groups. This may be useful when considering how very early identified ASD is similar or different from children with global developmental delay or language delay. **See *e*Figure 6**

© 2019 Pierce K et al. *JAMA Pediatrics.*

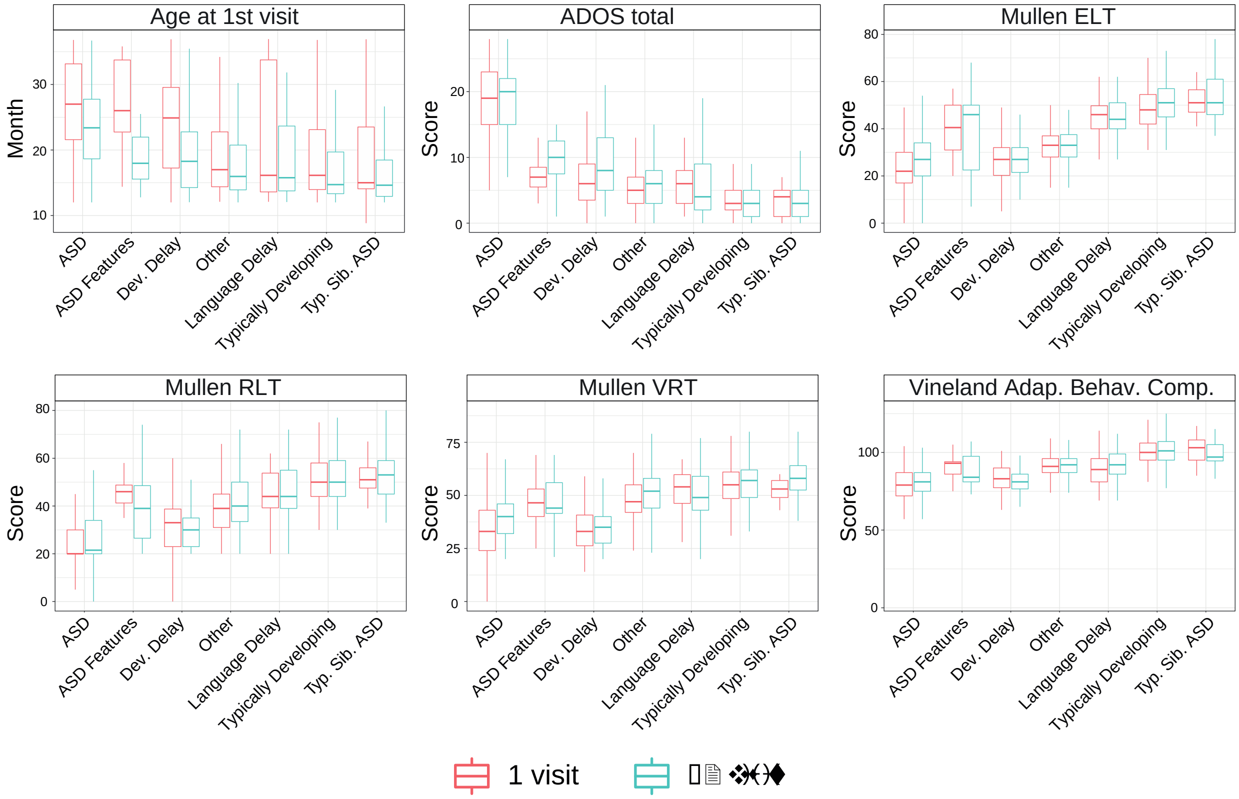

eFigure 1. Examination of clinical characteristics between excluded (1 visit) and included (≥ 2 visits) toddlers

As illustrated, toddlers with only one diagnostic visit tend to have their first visit at older ages. However, the two groups show similar clinical characteristics in broad set of clinical characteristics at their first diagnostic visit, suggesting minimal effect of selection bias in this regard.

© 2019 Pierce K et al. *JAMA Pediatrics.*

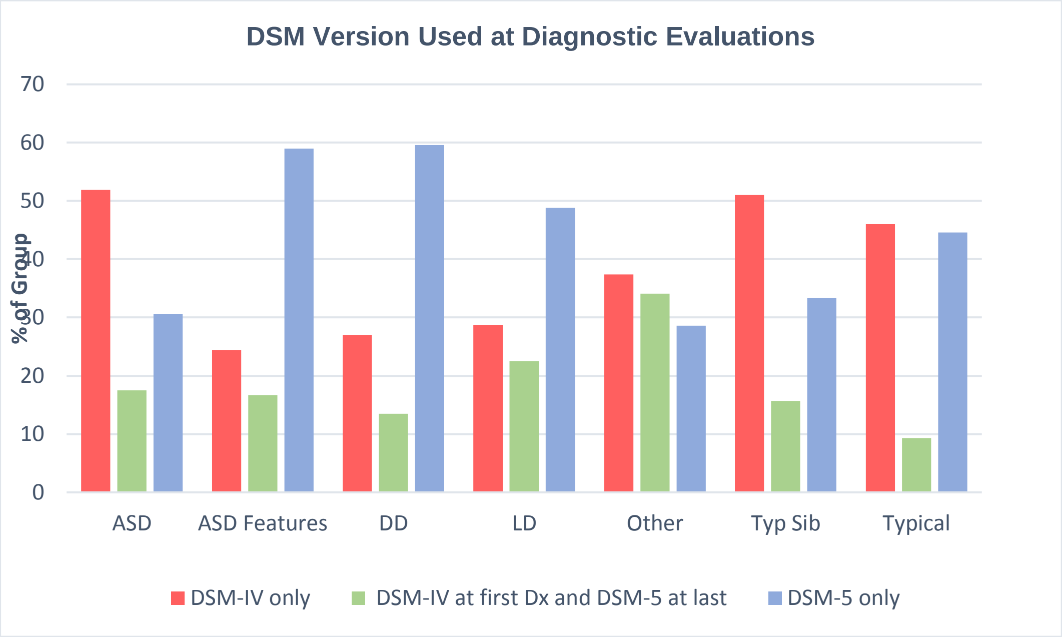

eFigure 2. Distribution of diagnostic judgements based on the DSM version
Bar graph illustrating the percentage of toddlers within each diagnostic group that received all of their diagnostic evaluations under DSM-IV criteria (red), DSM-5 criteria (blue) or both (light green).

© 2019 Pierce K et al. *JAMA Pediatrics.*

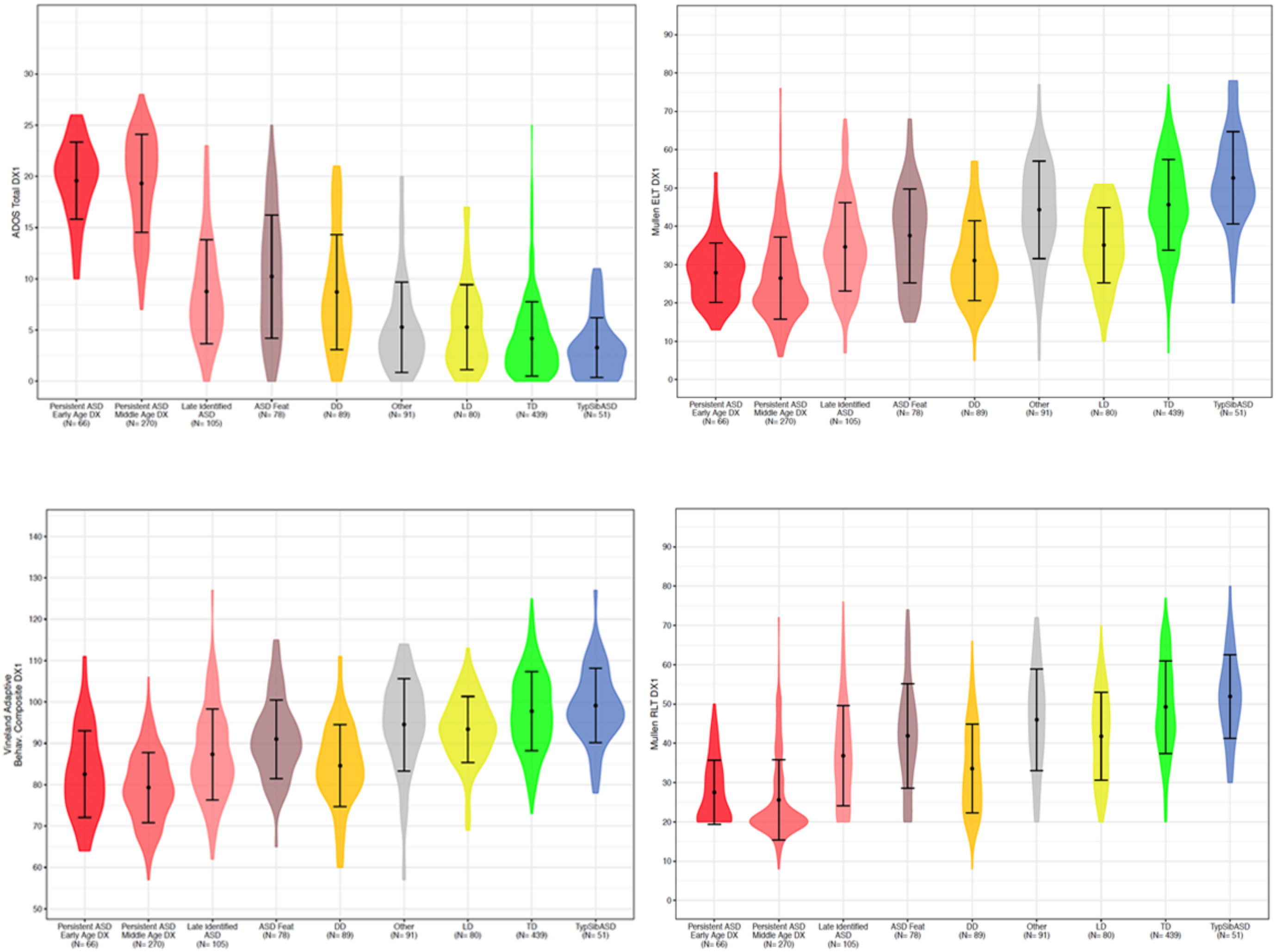

eFigure 3: Clinical characteristics of ASD group stratified by identification age and other DX groups

Violin plots illustrating clinical scores on the ADOS, Mullen expressive language subscale, Mullen receptive language subscale and the Vineland Adaptive Behavior Composite between toddlers with ASD who were identified between 12-18 months (Persistent ASD, Early Age DX), after 18 months (Persistent ASD, Middle Age DX), or who were not identified as ASD at their first diagnostic visit (Late Identified ASD) and all other diagnostic groups. This figure is an expanded version on Figure 4 in the main paper that only included TD toddlers with a persistent diagnosis of TD as a contrast group.   This expanded figure highlights the fact that toddlers with ASD that were initially missed by the clinician (i.e., “Late Identified” ASD) were showing delays at their first diagnostic evaluation visit. These delays, however, were similar to those exhibited by toddlers categorized as DD or LD, underscoring the challenges of differential diagnoses during early development.

© 2019 Pierce K et al. *JAMA Pediatrics.*

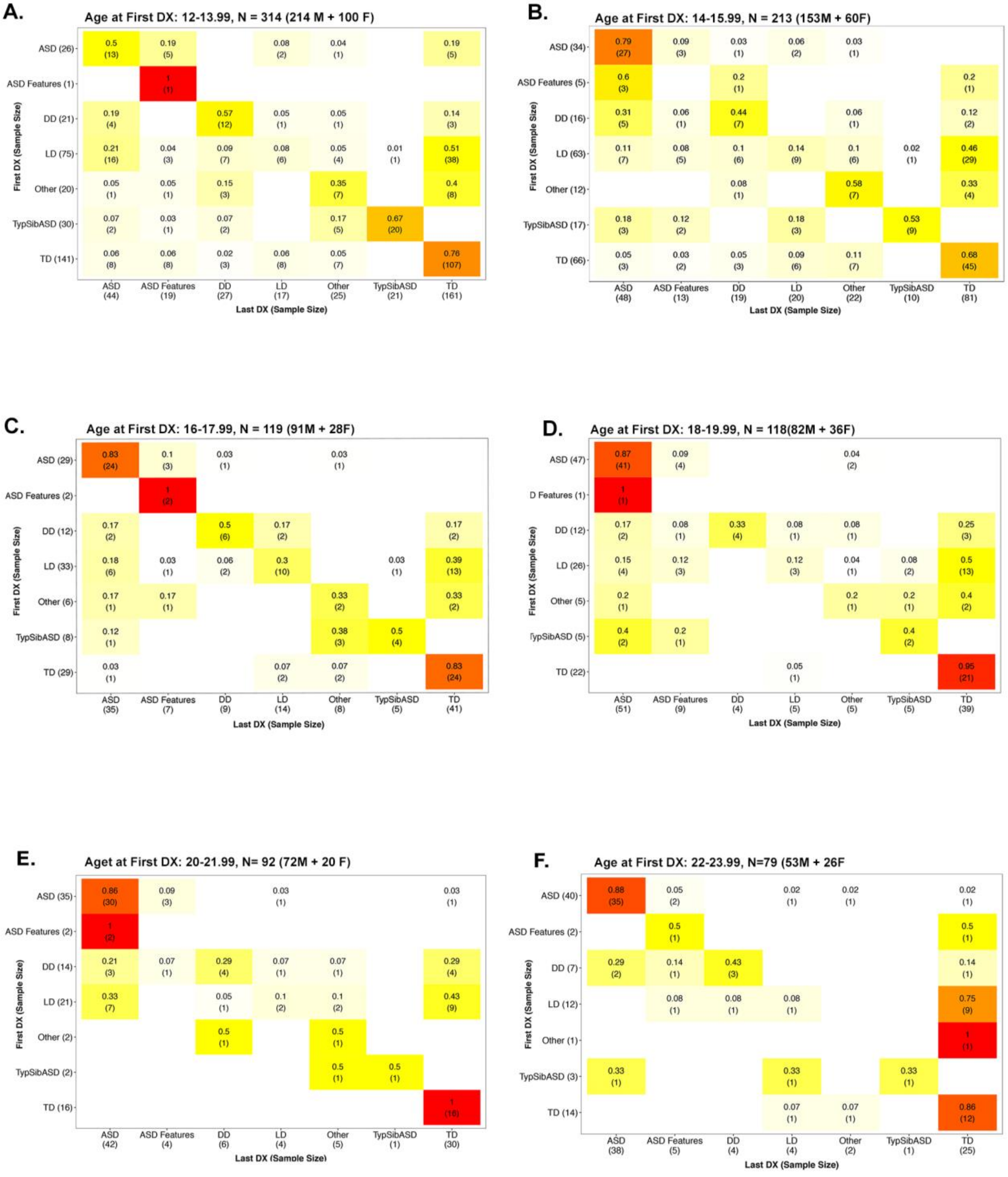

eFigure 4. Diagnostic Transition Tables within each 2-month age band

Panels A-M. Sample sizes for each age band are shown on the top of each table, N (M+ F). To see the diagnostic transition table for the full sample collapsed across all ages, see Figure 2 in the main manuscript.

Supplemental Figure Part II

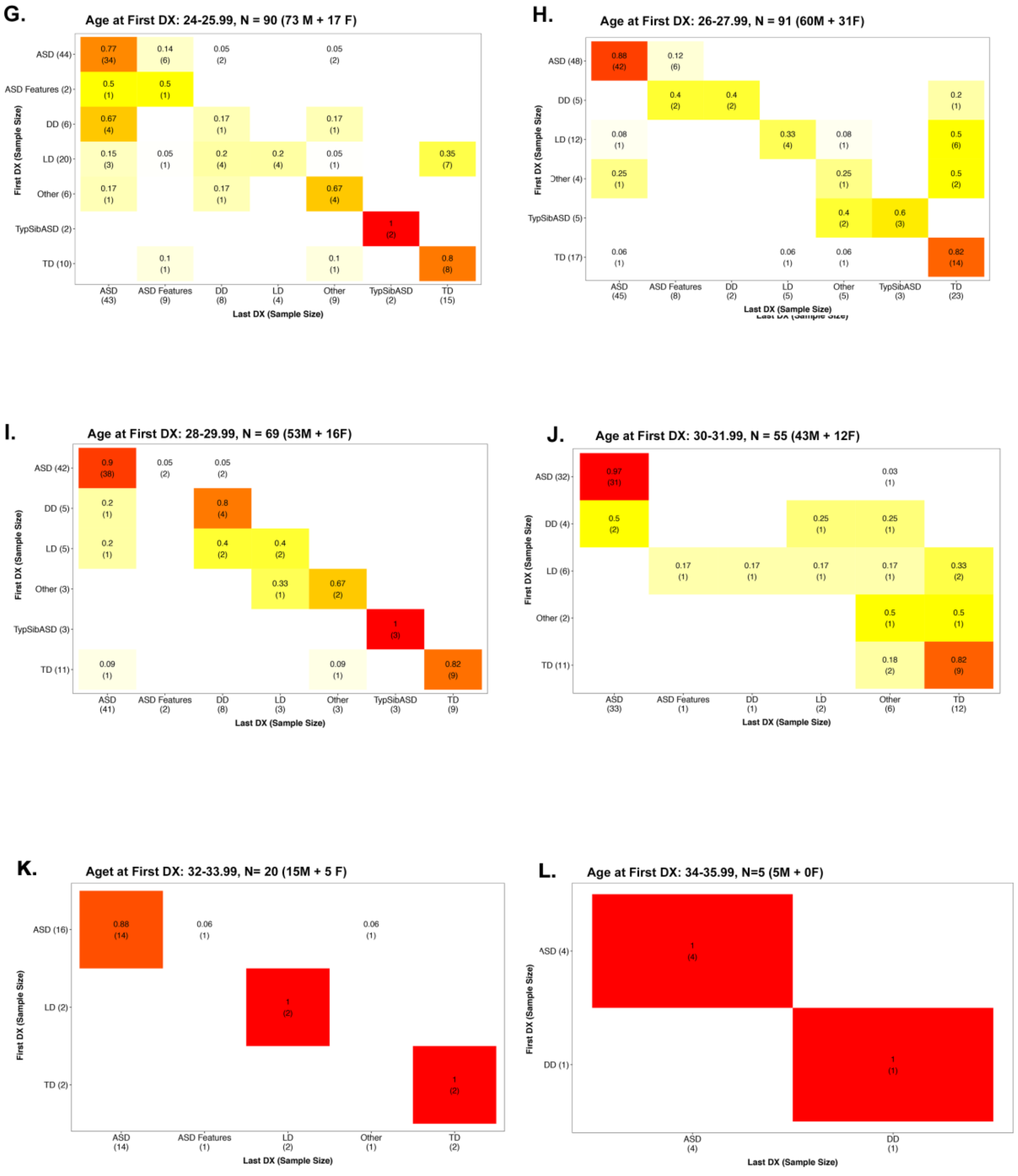

M. **Age at First DX: 36-36.99 N = 4 (4M + 0F) (Table Not Shown)**
3 ASD, DX stability coefficient = 1, 1 TD, DX stability cefficient = 1

eFigure 4 (continued). Diagnostic Transition Tables within each 2-month age band Panels A-M.
Sample sizes for each age band are shown on the top of each table, N (M+ F). To see the diagnostic transition table for the full sample collapsed across all ages, see Figure 2 in the main manuscript.

© 2019 Pierce K et al. *JAMA Pediatrics.*

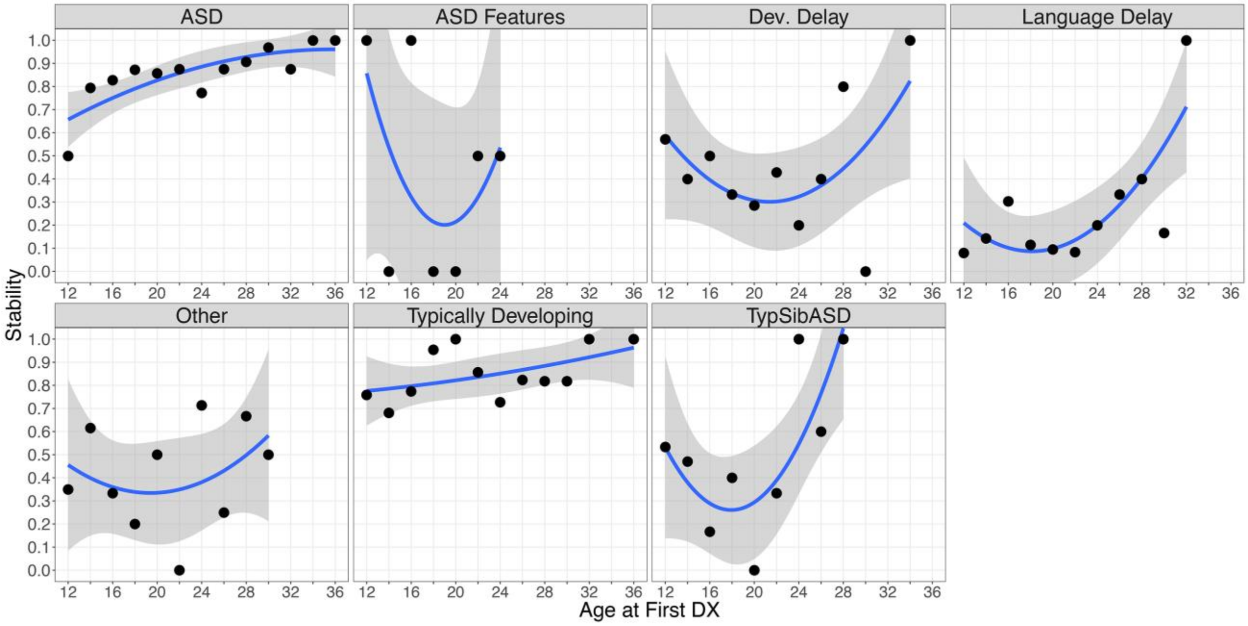

eFigure 5. Diagnostic stability plots by age at first diagnosis

Raw data plots illustrating diagnostic stability per group across 2-month age intervals based on the age at first diagnostic evaluation. Age intervals with missing data points reflects an absence of subjects who received their first diagnostic evaluation at that age. B-spline regression line is shown in blue; gray bands represent 95% confidence intervals for the fit line. Overall stability was highest in toddlers initially designated as ASD or TD as illustrated by the relatively tight confidence interval bands, and the largely consistent stability coefficients.

© 2019 Pierce K et al. *JAMA Pediatrics.*

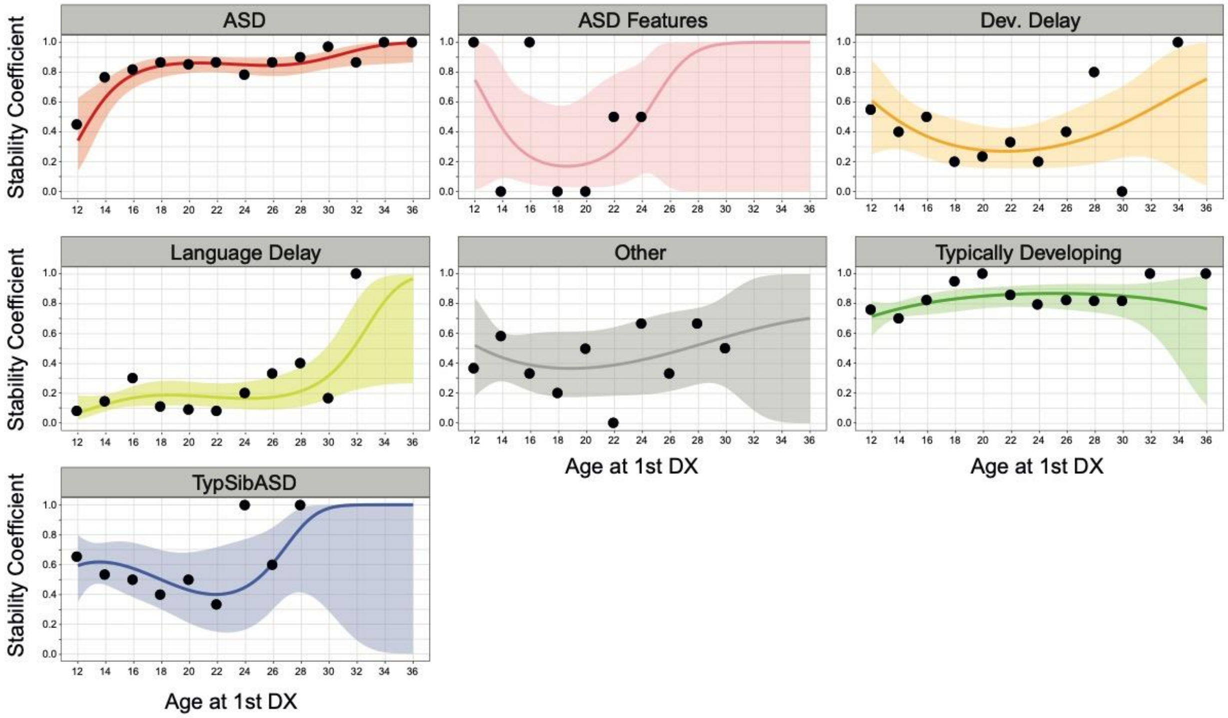

eFigure 6. Diagnostic stability after removing toddlers with non-verbal mental age < 12 mo

In our cohort, 73 toddlers had non-verbal mental age less than 12 months. Comparisons between this figure and Figure 3 in the main body of the paper illustrate that the removal of toddlers with non-verbal mental ages <12 months does not improve diagnostic stability at 12-months (stability actually declines, changing from .50 to .44). See also *e*Table 5.

© 2019 Pierce K et al. *JAMA Pediatrics.*

eTable 1. Overall logistic regression model

This model included four co-variates: first diagnosis, age at first diagnosis, gender, and time interval between first and last diagnostic visits. The contrast group associated with each covariate is designated as "Contr". Due to rounding, proportions may not add to one.

Co-variate		Odds Ratio	95% CI	Sample Size	Proportion (n_s/n)
	(Intercept)	9.9	6.21-16.08	NA	NA
First Diagnosis (Contr. ASD)	ASD Features	0.11	0.03-0.32	15	0.012
	Dev. Delay	0.15	0.09-0.25	103	0.08
	Language Delay	0.04	0.03-0.06	275	0.22
	Other	0.16	0.09-0.28	61	0.05
	Typically Developing	0.86	0.57-1.29	340	0.27
	Typ. Sib. ASD	0.35	0.2-0.62	75	0.06
Age at first diagnosis (Contr. ≥24)	<14	0.5	0.32-0.75	314	0.25
	14-17.99	0.69	0.46-1.04	289	0.23
	18-23.99	0.62	0.41-0.93	332	0.26
Gender (Contr. F)	Male	0.76	0.56-1.04	918	0.72
Time Interval	interval	0.99	0.98-1	1269	1

© 2019 Pierce K et al. *JAMA Pediatrics.*

eTable 2. Effect of age at first DX on stability coefficients

Statistics are based on within diagnostic group logistic regression models. Age group ≥24 was considered as the contrast. Statistics are presented as Odds Ratios, [95% CI].

	Covariate	ASD	ASD Features	Dev. Delay	Language Delay	Other	Typically Developing	Typ. Sib. ASD
	Intercept	7.22 [4.77-11.46]	1 [0.04-25.29]	0.62 [0.24-1.46]	0.41 [0.21-0.76]	1.14 [0.41-3.26]	4.78 [2.44-10.47]	4 [1-26.5]
Age at 1st DX (contr. ≥24)	<14	0.14 [0.06-0.34]	NA	2.17 [0.64-7.71]	0.21 [0.07-0.59]	0.47 [0.12-1.83]	0.66 [0.28-1.44]	0.5 [0.07-2.48]
	14-17.99	0.59 [0.28-1.3]	0.4 [0.01-13.62]	1.41 [0.45-4.57]	0.61 [0.27-1.39]	0.87 [0.22-3.48]	0.56 [0.23-1.26]	0.27 [0.04-1.35]
	18-23.99	0.92 [0.47-1.84]	0.25 [0-10.21]	0.81 [0.26-2.59]	0.28 [0.09-0.78]	0.29 [0.03-1.76]	3.42 [0.95-16.15]	0.17 [0.02-1.1]

© 2019 Pierce K et al. *JAMA Pediatrics.*

eTable 3. Stability coefficients within 2 months age bands.

Coefficients and associated confidence intervals (CIs) were estimated based on a logistic regression model in which non-linear effect of age was modeled using a B-spline method. Proportions (Prop.) columns indicate the proportion of toddlers with stable diagnosis at each age band. In age bands with no toddlers (n=0), the proportions are reported as NA. Statistics are in the format of Stability Coefficient [95% CI].

Age at 1st DX	ASD Stability Coef.	ASD Prop. (n_s/n)	ASD Features Stability Coef.	ASD Features Prop. (n_s/n)	Dev. Delay Stability Coef.	Dev. Delay Prop. (n_s/n)	Language Delay Stability Coef.	Language Delay Prop. (n_s/n)	Other Stability Coef.	Other Prop. (n_s/n)	Typically Developing Stability Coef.	Typically Developing Prop. (n_s/n)	Typ. Sib. ASD Stability Coef.	Typ. Sib. ASD Prop. (n_s/n)
**12-13.99**	.54 [.37-.71]	.50 [.32-.69]	.58 [.05-.97]	1 [0-1]	.53 [.35-.70]	.57 [.36-.77]	.09 [.05-.17]	.08 [.03-.15]	.43 [.23-.65]	.35 [.17-.57]	.74 [.66-.80]	.76 [.68-.82]	.62 [.47-.75]	.67 [.49-.82]
**14-15.99**	.76 [.66-.83]	.79 [.64-.91]	.30 [.07-.70]	0 [0-1]	.48 [.35-.62]	.44 [.22-.68]	.15 [.10-.21]	.14 [.07-.24]	.41 [.25-.58]	.58 [.31-83]	.78 [.71-.84]	.68 [.56-.79]	.60 [.42-.76]	.53 [.30-.75]
**16-17.99**	.84 [.76-.90]	.83 [.67-.93]	.19 [.04-.60]	1 [0-1]	.43 [.29-.58]	.50 [.24-.76]	.18 [.12-.27]	.30 [.17-47]	.39 [.20-.62]	.33 [.07-72]	.81 [.73-.88]	.83 [.67-93]	.54 [.34-.72]	.50 [.19-.81]
**18-19.99**	.87 [.80-.91]	.87 [.76-.95]	.17 [.03-.59]	0 [0-1]	.38 [.25-.53]	.33 [.12-.61]	.19 [.12-.28]	.12 [.03-.27]	.38 [.18-.62]	.20 [.01-.63]	.84 [.75-.90]	.95 [.81-1]	.46 [.25-.68]	.40 [.08-.80]
**20-21.99**	.87 [.81-.91]	.86 [.72-.95]	.22 [.03-.73]	0 [0-1]	.34 [.22-.49]	.29 [.10-.55]	.18 [.11-.27]	.10 [.02-.26]	.38 [.18-.62]	.50 [.04-.96]	.86 [.77-.91]	1 [0-1]	.40 [.17-.69]	.50 [.04-.96]

© 2019 Pierce K et al. *JAMA Pediatrics.*

Age at 1st DX	ASD		ASD Features		Dev. Delay		Language Delay		Other		Typically Developing		Typ. Sib. ASD	
	Stability Coef.	Prop. (n_s/n)	Stability Coef.	Prop. (n_s/n)	Stability Coef.	Prop. (n_s/n)	Stability Coef.	Prop. (n_s/n)	Stability Coef.	Prop. (n_s/n)	Stability Coef.	Prop. (n_s/n)	Stability Coef.	Prop. (n_s/n)
**22-23.99**	.86 [.80-.90]	.88 [.75-.95]	.37 [.07-.82]	.50 [.04-.96]	.32 [.19-.48]	.43 [.13-.77]	.17 [.10-.27]	.08 [0-.32]	.38 [.18-.65]	0 [0-1]	.87 [.78-.92]	.86 [.62-.97]	.41 [.14-.74]	.33 [.02-.84]
**24-25.99**	.84 [.78-.89]	.77 [.64-.88]	.64 [.10-.97]	.50 [.04-.96]	.31 [.17-.50]	.17 [.01-.55]	.17 [.09-.29]	.20 [.07-.40]	.41 [.18-.68]	.67 [.28-.94]	.87 [.77-.93]	.80 [.50-.96]	.52 [.22-.80]	1 [0-1]
**26-27.99**	.85 [.77-.90]	.88 [.76-.95]	.88 [0-1]	NA	.33 [.17-.55]	.40 [.08-.80]	.19 [.10-.34]	.33 [.12-.61]	.45 [.21-.71]	.25 [.02-.72]	.87 [.76-.94]	.82 [.60-.95]	.73 [.39-.92]	.60 [.20-.92]
**28-29.99**	.88 [.81-.92]	.90 [.79-.97]	.98 [0-1]	NA	.38 [.19-.62]	.80 [.37-.99]	.26 [.13-.44]	.4 [.08-.80]	.52 [.25-.78]	.67 [.16-.98]	.86 [.73-.94]	.82 [.54-.97]	.93 [.37-1]	1 [0-1]
**30-31.99**	.93 [.84-.97]	.97 [.87-1]	1 [0-1]	NA	.48 [.21-.76]	0 [0-1]	.42 [.20-.67]	.17 [.01-.55]	.60 [.16-.92]	.50 [.04-.96]	.85 [.68-.94]	.82 [.54-.97]	.99 [.17-1]	NA
**32-33.99**	.97 [.86-.99]	.88 [.66-.98]	1 [0-1]	NA	.62 [.17-.93]	NA	.69 [.25-.94]	1 [0-1]	.71 [.04-.99]	NA	.82 [.51-.95]	1 [0-1]	1 [.03-1]	NA

© 2019 Pierce K et al. *JAMA Pediatrics.*

Age at 1st DX	ASD		ASD Features		Dev. Delay		Language Delay		Other		Typically Developing		Typ. Sib. ASD	
	Stability Coef.	Prop. (n_s/n)	Stability Coef.	Prop. (n_s/n)	Stability Coef.	Prop. (n_s/n)	Stability Coef.	Prop. (n_s/n)	Stability Coef.	Prop. (n_s/n)	Stability Coef.	Prop. (n_s/n)	Stability Coef.	Prop. (n_s/n)
**34-36**	.99 [.89-1]	1 [0-1]	1 [0-1]	NA	.78 [.10-.99]	1 [0-1]	.92 [.27-1]	NA	.81 [0-1]	NA	.77 [.23-.98]	1 [0-1]	1 [0-1]	NA

© 2019 Pierce K et al. *JAMA Pediatrics.*

eTable 4. Overall age adjusted and unadjusted stability coefficients

Both adjusted and unadjusted coefficients were determined based on within diagnostic group logistic regression models. Overall, unadjusted stability coefficients were obtained by a logistic regression without considering age as a co-variate. Age adjusted stability coefficients are based on a logistic regression model in which non-linear effect of age was modeled using a B-splines method and are based on the median age at 1st diagnosis of our cohort across diagnosis groups (i.e., age of 17.6 months).

Diagnosis at 1st visit	Age Adjusted Stability Coefficients	Unadjusted Stability Coefficients
ASD	0.85 [0.78-0.9]	0.84 [0.8-0.87]
Dev. Delay	0.41 [0.28-0.57]	0.43 [0.33-0.52]
Language Delay	0.19 [0.12-0.28]	0.16 [0.12-0.21]
Other	0.38 [0.19-0.62]	0.43 [0.31-0.55]
ASD Features	0.18 [0.03-0.58]	0.33 [0.13-0.58]
Typically Developing	0.82 [0.73-0.88]	0.79 [0.74-0.83]
Typ. Sib. ASD	0.51 [0.31-0.71]	0.6 [0.49-0.71]

© 2019 Pierce K et al. *JAMA Pediatrics.*

eTable 5. Distribution of toddlers with non-verbal age below 12 mo. across diagnosis groups

Data is stratified by age at first diagnosis (DX) and the number of subjects with a VR score <12 months is denoted in the corresponding cell. In an exploratory analysis to determine the impact of VR score on ADOS results, subjects with a VR score < 12 months were removed (*e*Figure 2).

Age at First DX (mo)

Age Band	**12-13**	**14-15**	**16-17**	**18-19**	**20-21**	**22-23**	**24-25**	**26-27**	**28-29**	**30-31**	**32-33**	**34-35**	**36-37**	**Total**
**ASD**	9	7	4	3	3	2	2	2	1	0	1	0	0	34
**ASD Features**	1	0	0	0	0	0	0	0	0	0	0	0	0	1
**DD**	10	7	2	2	1	1	1	0	0	0	0	0	0	24
**LD**	0	0	0	0	0	0	0	0	0	0	0	0	0	0
**TD**	6	0	0	0	0	0	0	0	0	0	0	0	0	6
**Other**	3	0	3	0	0	0	1	0	0	0	0	0	0	7
**Typ. Sib**	1	0	0	0	0	0	0	0	0	0	0	0	0	1

© 2019 Pierce K et al. *JAMA Pediatrics.*

eTable 6. ASD subgroup between-group difference for all clinical domains

FirstGroup	SecondGroup	Attribute	tStatistics	tPval	tCIlow	tCIhigh	cohenD	cohenDlow	cohenDhigh
Persistent ASD Middle Age DX (N= 270)	Persistent ASD	ados_CoSoTotRRTot_1	-0.481705389	0.630879055	-1.346329603	0.81934977	-0.057355308	-0.327601884	0.212891267
Persistent ASD Middle Age DX (N= 270)	Persistent ASD	mullen_ELT_1	-1.890720521	0.060675266	-4.565290507	0.10139505	-0.20616518	-0.476830456	0.064500096
Persistent ASD Middle Age DX (N= 270)	Persistent ASD	mullen_RLT_1	-1.509171081	0.133965169	-4.387912691	0.59271161	-0.184858288	-0.455434661	0.085718085
Persistent ASD Middle Age DX (N= 270)	Persistent ASD	mullen_ELC_Std_1	-2.767362624	0.006544328	-8.516684463	-1.4127962	-0.331942902	-0.603329313	-0.060556492
Persistent ASD Middle Age DX (N= 270)	Persistent ASD	vine_AdapBehav_DomStd_1	-2.299001563	0.023897519	-5.958031534	-0.4328663	-0.358306004	-0.629885974	-0.086726034
Persistent ASD Middle Age DX (N= 270)	Late Identified	ados_CoSoTotRRTot_1	18.42041235	2.73E-43	9.437201409	11.7016196	2.175848916	1.90076496	2.450932871
Persistent ASD Middle Age DX (N= 270)	Late Identified	mullen_ELT_1	-6.785696352	1.46E-10	-11.61736291	-6.3841949	-0.783996935	-1.017182935	-0.550810935
Persistent ASD Middle Age DX (N= 270)	Late Identified	mullen_RLT_1	-8.290619193	4.00E-14	-14.41832141	-8.871252	-1.035081756	-1.273276459	-0.796887053
Persistent ASD Middle Age DX (N= 270)	Late Identified	mullen_ELC_Std_1	-8.981102751	5.89E-16	-22.21836757	-14.209814	-1.112780299	-1.35277902	-0.872781578
Persistent ASD Middle Age DX (N= 270)	Late Identified	vine_AdapBehav_DomStd_1	-6.671481159	4.25E-10	-10.31238749	-5.6005883	-0.858540662	-1.093079858	-0.624001466

© 2019 Pierce K et al. *JAMA Pediatrics.*

Persistent ASD Middle Age DX (N= 270)	TD	ados_CoSoTotRRTot_1	49.12761656	1.12E-174	15.62031688	16.9224341	4.236083894	3.931051757	4.54111603
Persistent ASD Middle Age DX (N= 270)	TD	mullen_ELT_1	-30.3806509	1.39E-114	-27.86358655	-24.478421	-2.619605733	-2.850475591	-2.388735875
Persistent ASD Middle Age DX (N= 270)	TD	mullen_RLT_1	-31.01763346	1.36E-121	-29.35824417	-25.861087	-2.674530269	-2.907647644	-2.441412894
Persistent ASD Middle Age DX (N= 270)	TD	mullen_ELC_Std_1	-36.7117478	4.81E-144	-45.95920825	-41.289862	-3.165511671	-3.419729912	-2.911293429
Persistent ASD Middle Age DX (N= 270)	TD	vine_AdapBehav_DomStd_1	-28.83471471	5.51E-111	-23.17715654	-20.220613	-2.486305649	-2.711827233	-2.260784065
Persistent ASD Early Age DX (N= 66)	Persistent ASD	ados_CoSoTotRRTot_1	0.481705389	0.630879055	-0.819349768	1.3463296	0.057355308	-0.212891267	0.327601884
Persistent ASD Early Age DX (N= 66)	Persistent ASD	mullen_ELT_1	1.890720521	0.060675266	-0.101395047	4.56529051	0.20616518	-0.064500096	0.476830456
Persistent ASD Early Age DX (N= 66)	Persistent ASD	mullen_RLT_1	1.509171081	0.133965169	-0.59271161	4.38791269	0.184858288	-0.085718085	0.455434661
Persistent ASD Early Age DX (N= 66)	Persistent ASD	mullen_ELC_Std_1	2.767362624	0.006544328	1.412796217	8.51668446	0.331942902	0.060556492	0.603329313
Persistent ASD Early Age DX (N= 66)	Persistent ASD	vine_AdapBehav_DomStd_1	2.299001563	0.023897519	0.432866292	5.95803153	0.358306004	0.086726034	0.629885974
Persistent ASD Early Age DX (N= 66)	Late Identified	ados_CoSoTotRRTot_1	15.98978314	2.63E-35	9.495190747	12.1706101	2.350336752	1.951453093	2.749220411

© 2019 Pierce K et al. *JAMA Pediatrics.*

Persistent ASD Early Age DX (N= 66)	Late Identified	mullen_ELT_1	-4.586490904	8.77E-06	-9.682333884	-3.8553285	-0.659975621	-0.977976885	-0.341974356
Persistent ASD Early Age DX (N= 66)	Late Identified	mullen_RLT_1	-5.911356824	1.85E-08	-13.00244983	-6.4919225	-0.854773859	-1.178018531	-0.531529188
Persistent ASD Early Age DX (N= 66)	Late Identified	mullen_ELC_Std_1	-5.628360872	7.47E-08	-17.89661977	-8.6020815	-0.810982785	-1.132939165	-0.489026406
Persistent ASD Early Age DX (N= 66)	Late Identified	vine_AdapBehav_DomStd_1	-2.836438586	0.005222941	-8.078902197	-1.4431757	-0.440554141	-0.754199089	-0.126909193
Persistent ASD Early Age DX (N= 66)	TD	ados_CoSoTotRRTot_1	33.78995575	2.06E-49	15.56118211	17.5085487	5.784751865	5.268729257	6.300774473
Persistent ASD Early Age DX (N= 66)	TD	mullen_ELT_1	-22.22026893	2.03E-41	-26.07532715	-21.802785	-2.929081357	-3.279173602	-2.578989112
Persistent ASD Early Age DX (N= 66)	TD	mullen_RLT_1	-20.7305243	6.16E-40	-28.16984161	-23.254288	-2.62234012	-2.958092851	-2.286587388
Persistent ASD Early Age DX (N= 66)	TD	mullen_ELC_Std_1	-22.93811597	9.74E-41	-42.00528147	-35.314308	-3.245783237	-3.611650633	-2.87991584
Persistent ASD Early Age DX (N= 66)	TD	vine_AdapBehav_DomStd_1	-13.2119346	9.33E-23	-21.28593211	-15.72094	-1.996134606	-2.306016365	-1.686252846

© 2019 Pierce K et al. *JAMA Pediatrics.*

© 2019 Pierce K et al. *JAMA Pediatrics.*

eReferences

1. Pierce K, Carter C, Weinfeld M, et al. Detecting, Studying, and Treating Autism Early: The One-Year Well-Baby Check-Up Approach. *Journal of Pediatrics.* 2011 159:458-465.
2. Broder Fingert S, Carter A, Pierce K, et al. Implementing systems-based innovations to improve access to early screening, diagnosis, and treatment services for children with autism spectrum disorder: An Autism Spectrum Disorder Pediatric, Early Detection, Engagement, and Services network study. *Autism.* 2018:1362361318766238.
3. Wetherby A, Prizant B. Communication and symbolic behavior scales developmental profile - first normed edition. Baltimore, MD: Paul H. Brookes; 2002.
4. Woolfenden S, Sarkozy V, Ridley G, Williams K. A systematic review of the diagnostic stability of Autism Spectrum Disorder. *Research in Autism Spectrum Disorders.* 2012;6:345-354.

© 2019 Pierce K et al. *JAMA Pediatrics.*
